# “Positive” Results Increase Down the Hierarchy of the Sciences

**DOI:** 10.1371/journal.pone.0010068

**Published:** 2010-04-07

**Authors:** Daniele Fanelli

**Affiliations:** INNOGEN and ISSTI-Institute for the Study of Science, Technology & Innovation, The University of Edinburgh, Edinburgh, United Kingdom; University of East Piedmont, Italy

## Abstract

The hypothesis of a Hierarchy of the Sciences with physical sciences at the top, social sciences at the bottom, and biological sciences in-between is nearly 200 years old. This order is intuitive and reflected in many features of academic life, but whether it reflects the “hardness” of scientific research—i.e., the extent to which research questions and results are determined by data and theories as opposed to non-cognitive factors—is controversial. This study analysed 2434 papers published in all disciplines and that declared to have tested a hypothesis. It was determined how many papers reported a “positive” (full or partial) or “negative” support for the tested hypothesis. If the hierarchy hypothesis is correct, then researchers in “softer” sciences should have fewer constraints to their conscious and unconscious biases, and therefore report more positive outcomes. Results confirmed the predictions at all levels considered: discipline, domain and methodology broadly defined. Controlling for observed differences between pure and applied disciplines, and between papers testing one or several hypotheses, the odds of reporting a positive result were around 5 times higher among papers in the disciplines of Psychology and Psychiatry and Economics and Business compared to Space Science, 2.3 times higher in the domain of social sciences compared to the physical sciences, and 3.4 times higher in studies applying behavioural and social methodologies on people compared to physical and chemical studies on non-biological material. In all comparisons, biological studies had intermediate values. These results suggest that the nature of hypotheses tested and the logical and methodological rigour employed to test them vary systematically across disciplines and fields, depending on the complexity of the subject matter and possibly other factors (e.g., a field's level of historical and/or intellectual development). On the other hand, these results support the scientific status of the social sciences against claims that they are completely subjective, by showing that, when they adopt a scientific approach to discovery, they differ from the natural sciences only by a matter of degree.

## Introduction

Although it is still controversial, the idea of a Hierarchy of the Sciences is nearly 200 years old [1], [2], [3]. Philosopher and historian of science August Comte (1798–1857) first suggested that scientific disciplines differed systematically in the complexity and generality of their subject of study, in the precision with which these subjects are known, and in their level of intellectual and historical development. Comte hypothesised a rank order in which what he called “celestial physics” (astronomy) preceded “terrestrial physics” (physics and chemistry), followed by “organic physics” (biology) and “social physics” (which he later renamed sociology) [1], [4]. Comte believed that sociology was the queen of all disciplines and the ultimate goal of all research, but also the most complex and least developed of the sciences [4].

Similar ideas have been proposed by contemporaries of Comte (e.g. William Whewell [5]) and by modern philosophers and sociologists of science who, for example, have distinguished between “hard” and “soft” sciences [6], [7], different levels of “empiricism” [8], different levels of “codification” [9], “pre- and post-paradigmatic” sciences [10], and argued that fields of research differ in the level of agreement on a single set of theories and methodologies [10], the rigour with which data is related to theory [7], the extent to which the choice of problems and decisions made in solving problems are based upon cognitive as opposed to non-cognitive criteria [11], the level of “consensus on the significance of new knowledge and the continuing relevance of old” [9], their explanatory success [12]. These scholars did not always endorse the exact same definitions and hierarchies, but they all shared an intuition that here we will summarize as follows: in some fields of research (which we will henceforth indicate as “harder”) data and theories speak more for themselves, whereas in other fields (the “softer”) sociological and psychological factors – for example, scientists' prestige within the community, their political beliefs, their aesthetic preferences, and all other non-cognitive factors – play a greater role in all decisions made in research, from which hypothesis should be tested to how data should be collected, analyzed, interpreted and compared to previous studies.

The hypothesised Hierarchy of the Sciences (henceforth HoS) is reflected in many social and organizational features of academic life. When 222 scholars rated their perception of similarity between academic disciplines, results showed a clustering along three main dimensions: a “hard/soft” dimension, which roughly corresponded to the HoS; a “pure/applied” dimension, which reflected the orientation of the discipline towards practical application; and a “life/non-life” dimension [13]. These dimensions have been validated by many subsequent studies, which compared disciplines by parameters including: average publication rate of scholars, level of social connectedness, level of job satisfaction, professional commitment, approaches to learning, goals of academic departments, professional duties of department heads, financial reward structures of academic departments, and even response rates to survey questionnaires [14], [15], [16], [17].

Numerous studies have taken a direct approach, and have attempted to compare the hardness of two or more disciplines, usually psychology or sociology against one or more of the natural sciences. These studies used a variety of proxy measures including: ratio of theories to laws in introductory textbooks, number of colleagues acknowledged in papers, publication cost of interrupting academic career for one year, proportion of under 35 s who received above-average citations, concentration of citations in the literature, rate of pauses in lectures given to undergraduates, immediacy of citations, anticipation of one's work by colleagues, average age when receiving the Nobel prize, fraction of journals' space occupied by graphs (called Fractional Graph Area, or FGA), and others [17], [18]. According to a recent review, some of these measures are correlated to one-another and to the HoS [2]. One parameter, FGA, even appears to capture the relative hardness of sub-disciplines: in psychology, FGA is higher in journals rated as “harder” by psychologists, and also in journals specialised in animal behaviour rather than human behaviour [19], [20], [21].

Whether disciplines really differ in hardness and can be ranked accordingly, however, is still controversial [3], [12], [21], [22]. This controversy is manifest, for example, in the debate on the applicability of the scientific method within disciplines like psychology or sociology. At one extreme are researchers that approach the social sciences like any other and test hypotheses through laboratory and field experiments; at the other extreme, eminent scholars argue that the social sciences are qualitatively different from other disciplines and that scientific objectivity within them is purely a myth [23], [24], [25], [26], [27]. Radically anti-hierarchy positions have been developed within the “second wave” of science studies and its “postmodern” derivations, according to which all scientific knowledge is “socially constructed” and thus not different from any other form of knowledge, faith or politics [28], [29]. Under this perspective, all the empirical measures of hardness listed above could be re-interpreted as just reflecting cultural differences between “academic tribes” [30].

Several lines of evidence support a non-hierarchical view of the sciences. The consensus between scientists within a field, measured by several independent parameters including level of agreement in evaluating colleagues and research proposals, is similar in physics and sociology [3]. The heterogeneity of effect sizes in meta-analyses also appears to be similar in the physical and the social sciences, suggesting a similar level of empirical cumulativeness [22]. Historical reconstructions show that scientific controversies are common at the frontier of all fields, and the importance and validity of experiments is usually established in hindsight, after a controversy has settled [31], [32]. Analysis of molecular biology papers showed that the interpretation of experiments is heavily influenced by previously published statements, regardless of their verity [33]. In evolutionary biology, published estimates on the heritability of sexually selected traits in various species were low for many years, but then suddenly increased when new mathematical models predicted that heritability should be high [34]. Cases of “pathological science”, in which a wrong theory or non-existent phenomenon are believed for many years and are “supported” by empirical data, have been observed in all fields, from parapsychology to physics [35].

The contrast between indirect measures of hardness, which point to a hierarchy, and evidence of high controversy and disagreement in all kinds of research has inspired an intermediate position, which distinguishes between the “core” and the “frontier” of research. The core is the corpus of agreed upon theories and concepts that researchers need to know in order to contribute to the field. Identifiable with the content of advanced university textbooks, the core is clearly more developed and structured in the physical than in the social sciences [11], [36]. The frontier is where research is actually done, where scientists produce new data and concepts, most of which will eventually be contradicted or forgotten and will never make it to the core. At the frontier, levels of uncertainty and disagreement might be similar across fields [3], [36].

The question, therefore, is still unanswered: does a Hierarchy of the Sciences really exist? Does the hardness of research vary systematically across disciplines? This study compared scientific papers at the frontier of all disciplines using an intuitive proxy of bias. Papers that declared to have tested a hypothesis were sampled at random from all 10837 journals in the Essential Science Indicators database, which univocally classifies them in 22 disciplines. It was then determined whether the authors of each paper had concluded to have found a “positive” (full or partial) or a “negative” (no or null) support for the tested hypothesis. The frequency of positive and negative results was then compared between disciplines, domains and methodological categories. Papers were classified by discipline based on the journal in which they were published. Disciplinary categories (e.g. pure/applied, life/non-life, etc…) followed previous classifications based on the perception of scholars [13], [14], [15], [16], [17]. Methodological categories are based on very general characteristics of the object of study and the parameters measured in each paper. The term “methodology”, therefore, in this paper is used in its broadest possible sense of “system of methods and principles used in a particular discipline” [37].

Since papers were selected at random with respect to all factors, the proportion of positive results in this sample is a proxy of the level of confirmation bias. Scientists, like all other human beings, have an innate tendency to confirm their expectations and the hypotheses they test [38]. This confirmation bias, which operates largely at the subconscious level, can affect the collection, analysis, interpretation and publication of data [39], [40] and thus contribute to the excess of positive results that has been observed in many fields [38], [41], [42], [43], [44]. In theory, application of the scientific method should prevent these biases in all research. In practice, however, in fields where theories and methodologies are more flexible and open to interpretation, bias is expected to be higher [45].

In sum, if the HoS hypothesis is correct, scientists in harder fields should accept more readily any result their experiments yield, while those in softer fields should have more freedom to choose which theories and hypotheses to test and how to analyze and interpret their own and their colleagues' results. This freedom should increase their chances to “find” in the data what they believe to be true (see the Discussion section for a detailed analysis), which leads to the prediction that papers will report more negative results in the harder sciences than in the softer.

## Results

**Figure 1:**
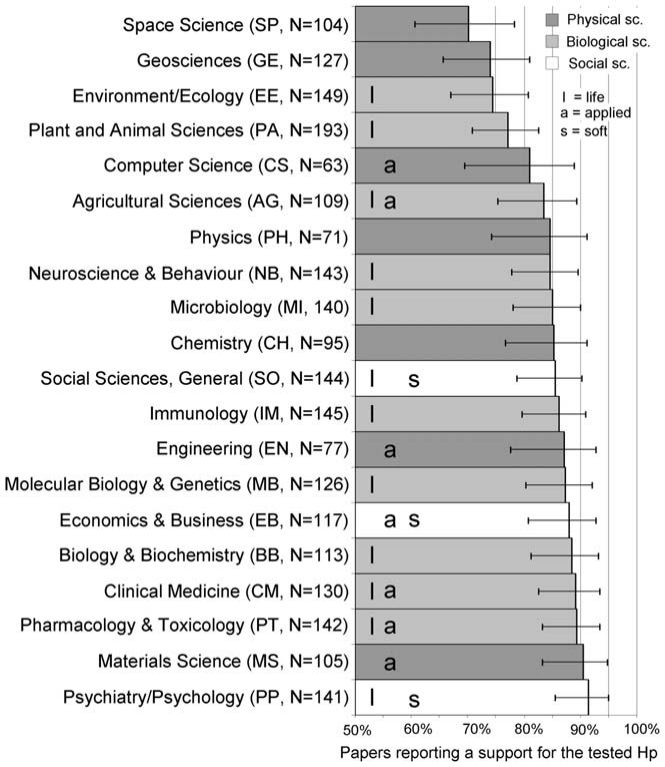
Positive Results by Discipline. Name of discipline, abbreviation used throughout the paper, sample size and percentage of “positive” results (i.e. papers that support a tested hypothesis). Classification by discipline was based on the Essential Science Indicators database, the hard/soft, pure/applied and life/non-life categories were based on previous literature (see text for details). Error bars represent 95% logit-derived confidence interval.

A total of 2434 papers were included in the analysis. No paper testing a hypothesis was retrieved from mathematical journals, and the “multidisciplinary” category (which includes journals like Nature, Science, PNAS, etc…) was excluded. Therefore, the sample represented 20 of the 22 disciplines in the Essential Science Indicators database (Fig. 1). Overall, 2045 papers (84%) reported a positive or partial support for the tested hypothesis. Positive results were distributed non-randomly between disciplines (*X* ^2^ = 61.934. df = 19, p<0.0001).

### Negative results by discipline, dimension and domain

Space Science had the lowest percentage of positive results (70.2%) and Psychology and Psychiatry the highest (91.5%). The overlap between disciplines in the physical, biological and social sciences was considerable (Fig. 1), yet the rank observed (based on the frequency of positive results) and that predicted by the hypothesis (physical = I, biological = II and social sciences = III) tended to correlate when all disciplines were included (Kendall's τ-c = 0.353±0.194SE, T = 1.813, p = 0.07), and were significantly correlated when only pure disciplines [13], [14], [16] were included (τ-c = 0.568±0.184SE, T = 3.095, p = 0.002). Applied disciplines showed no significant trend (τ-c = 0.061±0.364SE, T = 0.168, p = 0.867).

**Figure 2:**
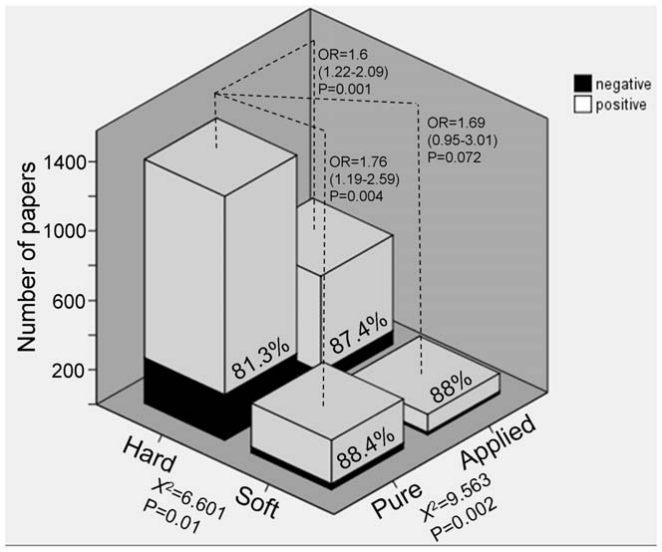
Positive Results by Disciplinary Dimension. Number of papers that supported (white) or failed to support (black) a tested hypothesis, classified by disciplinary categories based on dimensions identified by previous studies (see text for explanations). Percentage in each bar refers to positive results. OR = Odds Ratio (and 95%Confidence Interval) of reporting a positive result compared to the reference category of Hard/Pure disciplines. Chi square was calculated for each dimension separately (for category composition see Fig. 1).

Of the three disciplinary dimensions identified by previous studies [13], [14], [16], the hard/soft and the pure/applied dimensions were significantly associated with the frequency of positive results (Figure 2). The odds among soft disciplines were over 50% higher than among hard sciences (OR(95%CI) = 1.529(1.037–2.116), p = 0.011). The odds of reporting a positive result among papers published in hard-applied, soft-pure and soft-applied disciplines [13], [14], [16] were around 70% higher than among hard-pure disciplines (Fig. 2). The life/non-life dimension was not significantly associated with the frequency of positive results alone (*X* ^2^ = 2.675, p = 0.102; power to detect a small effect (Cohen's w = 0.1) = 0. 998), but it was when controlling for the other two dimensions (Wald = 5.493, p = 0.019, OR(95%CI) of life vs. non-life = 1.327(1.047-1.681)).

**Figure 3:**
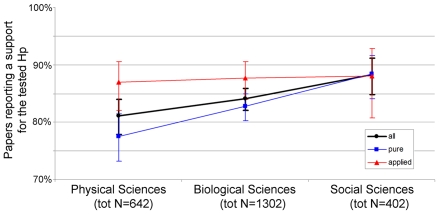
Positive Results by Disciplinary Domain. Percentage of papers that supported a tested hypothesis, classified by disciplinary domain. Blue = including only pure disciplines, Red = including only applied disciplines, Black = all disciplines included. Error bars represent 95% logit-derived confidence interval. For domain composition see Figure 4.

The disciplinary domain of a paper was a significant predictor of positive results when all disciplines were included (Wald = 9.335, df = 2, p = 0.009, OR(95%CI) of biological vs. physical sciences = 1.228(0.962-1.569), OR(95%CI) of social vs. physical sciences = 1.754(1.220-2.522)). When only pure disciplines were included, the effect was stronger (N = 1691, Wald = 13.34, p = 0.001, OR(95%CI) of biological vs. physical sciences = 1.387(1.041-1.847), OR(95%CI) or social vs. physical sciences = 2.207(1.431-3.402)). Among applied disciplines, however, positive results were uniformly high and not significantly different (N = 743, Wald = 0.110, p = 0.946; power to detect a small (OR = 1.5), medium (OR = 2.5) and large effect (OR = 4.5), respectively = 0.343, 0.89 and 0.996; OR(95%CI) of biological vs. physical sciences = 1.068(0.66-1.727), OR(95%CI) of social vs. physical = 1.105(0.565-2.161)) (Fig. 3).

### Negative results by methodological category

**Figure 4:**
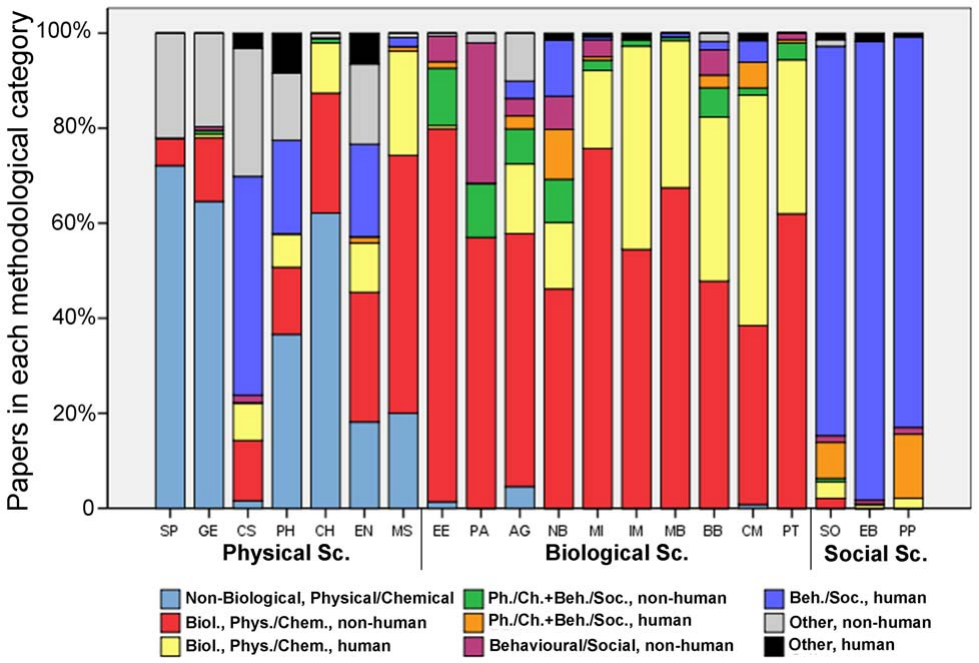
General Methodology by Discipline and by Domain. Methodology employed by papers in different disciplines and domains. Methodological categories correspond to basic characteristics of the outcome: whether it measured physical/chemical parameters as opposed to behavioural parameters, and whether the object of study was non-biological, biological non-human, or biological human (see Methods for further details).

The methodology of papers varied significantly between disciplines (*X* ^2^ = 4271.298, df = 152, p<0.001), but there was also considerable within-discipline variability, particular among the physical and biological sciences (Fig. 4).

**Figure 5:**
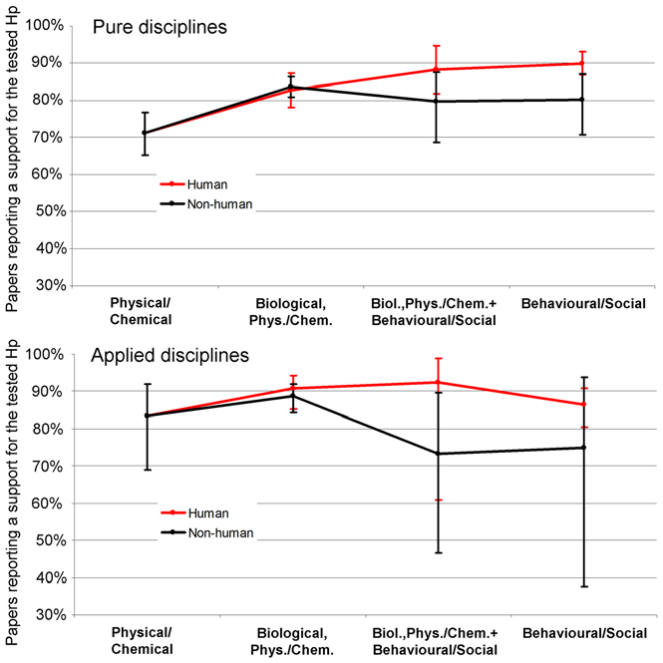
Positive Results by Methodological Category. Percentage of papers that supported a tested hypothesis in pure (top) and applied (bottom) disciplines, plotted by general characteristics of their methodology (defined by the outcome, see also Fig. 4). The “other methodology” category is not shown. Black = studies on non-human material or subjects, Red = studies on human material or subjects. Error bars represent 95% logit-derived confidence interval.

Methodological category was a significant predictor of positive results both when all disciplines and only pure disciplines were included (respectively, Wald = 37.943 and Wald = 33.834, df = 8, p<0.001), but not when only applied disciplines were included (Wald = 9.328, p = 0.315; power to detect a small, medium and large effect, respectively 0.18, 0.575 and 0.867) (Fig. 5). Including all disciplines, behavioural/social studies on humans (whether or not they included non-behavioural methods) reported significantly more positive results than behavioural studies on non-humans (tot N = 685, Wald = 9.669, df = 1, p = 0.002, OR(95%CI) = 2.046(1.303–3.213), while no difference between human and non-human was observed among biological, non-behavioural studies (tot N = 1328, Wald = 0.232, df = 1, p = 0.630, OR(95%CI) = 1.088(0.771–1.537); power to detect a small, medium and large effect, respectively = 0.551, 0.991 and 0.999). These latter reported significantly more positive results than behavioural studies on non-humans (tot N = 1511, Wald = 4.764, df = 1, p = 0.029, OR(95%CI) = 1.541(1.045–2.273).

### Confounding factors and corrected Odds-Ratios

Positive and negative results were not significantly associated with the five-year impact factor of the journal standardized by discipline (N = 2273, Student's t (equal variances not assumed) = -1.356, df = 511.827, p = 0.176; power to detect a small effect = 0.968), nor to the year of publication (*X* ^2^ = 11.413, df = 7, p = 0.122, Cramer's V = 0.068; power to detect a small effect = 0.97). Controlling for these two factors in regression models did not alter the results in any relevant way.

The frequency of negative results in papers that tested multiple hypotheses (N = 151, in which only the first hypothesis was considered), was significantly higher than in papers testing only one hypothesis (*X* ^2^ = 13.591, df = 1, p<0.001). Multiple-hypotheses papers were more frequent in the social than in the biological and the physical sciences (respectively, 18.47% (number of multiple papers N = 76), 4.46% (N = 62) and 1.87% (N = 12), *X* ^2^ = 140.308, df = 2, p<0.001, Cramer's V = 0.240), and were most frequent in the discipline of Economics and Business (47%, N = 55). However, the frequency of negative results in multiple-hypotheses papers was not significantly different between disciplines nor between disciplinary domains (respectively, *X* ^2^ = 15.567 df = 17, p = 0.555, V = 0.322, and *X* ^2^ = 4.303, df = 2, p = 0.116, V = 0.169), although only large effects can be excluded with significant confidence (power to detect a small, medium and large effect, respectively = 0.09, 0.59 and 0.98 for disciplines; 0.18, 0.92, 0.99 for domains).

When correcting for the confounding effect of presence/absence of multiple hypotheses, the odds of reporting a positive result were around five times higher for papers published in Psychology and Psychiatry and Economics and Business than in Space Science (Table 1, Nagelkerke R^2^_N_ = 0.051). When correcting for the confounding effect of pure/applied discipline and presence/absence of multiple hypotheses, the odds of reporting a positive results were about 2.3 times significantly higher for papers in the social sciences compared to the physical sciences (Table 2, R^2^_N_ = 0.030), and about 3.4 times significantly higher for behavioural and social studies on people compared to physical-chemical studies (Table 3, R^2^_N_ = 0.045).

**Table 1:** Logistic regression slope, standard error, Wald test with statistical significance, Odds Ratio and 95% Confidence Interval of the probability for a paper to report a positive result, depending on the following study characteristics: discipline of the journal in which the paper was published, papers testing more than one hypothesis (only the first of which was included in the study).

Variable	B	SE	Wald	df	Sig.	OR	95%CI OR
Discipline (all)			61.238	19	<0.001		
Geosciences	0.198	0.295	0.453	1	0.501	1.219	0.684–2.174
Environment/ Ecology	0.353	0.289	1.490		0.222	1.423	0.808–2.508
Plant and Animal Sciences	0.472	0.277	2.900	1	0.089	1.604	0.931–2.761
Computer Science	0.711	0.390	3.329	1	0.068	2.036	0.949–4.372
Agricultural Sciences	0.826	0.337	6.014	1	0.014	2.284	1.180–4.420
Physics	0.856	0.392	4.766	1	0.029	2.354	1.091–5.078
Neuroscience & Behaviour	0.872	0.316	7.616	1	0.006	2.393	1.288–4.446
Microbiology	0.903	0.320	7.973	1	0.005	2.467	1.318–4.616
Chemistry	0.911	0.360	6.391	1	0.011	2.487	1.227–5.041
Social Sciences, General	1.006	0.321	9.808	1	0.002	2.735	1.457–5.134
Immunology	0.984	0.323	9.311	1	0.002	2.676	1.422–5.035
Engineering	1.076	0.402	7.175	1	0.007	2.934	1.335–6.448
Mol. Biology & Genetics	1.081	0.343	9.930	1	0.002	2.947	1.505–5.772
Economics & Business	1.624	0.385	17.780	1	<0.001	5.073	2.385–10.792
Biology & Biochemistry	1.216	0.365	11.084	1	0.001	3.372	1.649–6.897
Clinical Medicine	1.286	0.355	13.090	1	<0.001	3.618	1.803–7.262
Pharm. & Toxicology	1.297	0.347	13.936	1	<0.001	3.658	1.851–7.226
Materials Science	1.395	0.396	12.433	1	<0.001	4.034	1.858–8.760
Psychiatry/Psychology	1.569	0.372	18.427	1	<0.001	4.935	2.381–10.230
Multiple hypotheses	−0.877	0.221	15.756	1	<0.001	0.416	0.27–0.642
Constant	0.856	0.214	15.962	1	<0.001	2.355	

The effect of discipline was tested for overall effect then each discipline was contrasted to Space Science by indicator contrast.

**Table 2:** Logistic regression slope, standard error, Wald test with statistical significance, Odds Ratio and 95% Confidence Interval of the probability for a paper to report a positive result, depending on the following study characteristics: disciplinary domain, papers testing more than one hypothesis (only the first of which was included in the study), and papers published in pure as opposed to applied disciplines.

Variable	B	SE	Wald	df	Sig.	OR	95%CI OR
Domain (all)			17.805	2	<0.001		
Biological sciences	0.297	0.127	5.487	1	0.019	1.346	1.05–1.726
Social sciences	0.813	0.194	17.519	1	<0.001	2.256	1.541–3.301
Multiple hypotheses	−1.036	0.207	25.11	1	<0.001	0.355	0.237–0.532
Pure discipline	−0.490	0.131	14.031	1	<0.001	0.613	0.474–0.792
Constant	1.803	0.136	176.034	1	<0.001	6.071	

Disciplinary domain was tested for overall effect, then biological and social sciences were each contrasted to physical sciences by indicator contrast.

**Table 3:** Logistic regression slope, standard error, Wald test with statistical significance, Odds Ratio and 95% Confidence Interval of the probability for a paper to report a positive result, depending on the following study characteristics: methodological category, papers testing more than one hypothesis (only the first of which was included in the study), and papers published in pure as opposed to applied disciplines.

Variable	B	SE	Wald	df	Sig.	OR	95%CI OR
Methodological category (all)			40.048	8	<0.001		
Biological, Ph/Ch, non-human	0.763	0.163	21.870	1	<0.001	2.145	1.558–2.954
Biological, Ph/Ch, human	0.750	0.205	13.449	1	<0.001	2.117	1.418–3.161
Ph/Ch+Beh/Soc, non-human	0.332	0.299	1.227	1	0.268	1.393	0.775–2.505
Ph/Ch+Beh/Soc, human	1.164	0.425	7.499	1	0.006	3.201	1.392–7.362
Behavioural/Social, non-human	0.497	0.287	2.991	1	0.084	1.643	0.936–2.885
Behavioural/Social, human	1.213	0.213	32.421	1	<0.001	3.364	2.215–5.107
Other, non human	0.469	0.284	2.738	1	0.098	1.599	0.917–2.788
Other, human	0.609	0.565	1.159	1	0.282	1.838	0.607–5.566
Multiple hypotheses	−1.058	0.209	25.756	1	<0.001	0.347	0.231–0.522
Pure discipline	−0.343	0.134	6.599	1	0.01	0.709	0.546–0.922
Constant	1.303	0.177	53.93	1	<0.001	3.682	

Methodological category (see text for details) was tested for overall effect, then each category was contrasted by indicator contrast to physical/chemical studies on non-biological material.

## Discussion

We analyzed a large sample of papers that, by declaring to have tested a hypothesis, had placed themselves at the research frontier of all disciplines and explicitly adopted the hypothetico-deductive method of scientific inquiry, with its assumptions of objectivity and rigour [24]. The frequency with which these papers reported a positive result was significantly predicted by the hardness (as it is perceived by scholars and suggested by numerous indirect measures) of their discipline, domain, and overall methodology.

These results must be generated by a combination of factors that, as will be discussed below, cannot be separated in this analysis. Overall, however, they support the existence of a Hierarchy of the Sciences, in which scientific rigour and objectivity are roughly inversely proportional to the complexity of subject matter and possibly other field-specific characteristics (e.g. level of development, see below). On the other hand, the differences observed were only a matter of degree. This supports the scientific status of the social sciences against claims that they are qualitatively different from the natural sciences and that a scientific method based on objectivity cannot be applied to them [25], [26], [27].

Not all observations matched the predicted hierarchy, however. At the disciplinary level, Psychology and Psychiatry had more positive results than Social Sciences, General, contradicting previous studies that placed psychology between biology and sociology [18], [21]. Moreover, Physics and Chemistry had more positive results than Social Sciences, General and a few biological disciplines. At the level of methodology, biological, non-behavioural studies on humans and non-humans had more positive results than behavioural studies on non-humans. At both levels, papers in applied disciplines showed a markedly different pattern, having uniformly high frequencies of positive results.

Overall, the predictive power of the regression models in this study was highly significant statistically, but never exceeded a 5.1% reduction in error (although the validity of R^2^-equivalents in logistic regression is controversial [46]). This value might appear small, but it is comparable to the average variance explained, for example, by ecological studies (which is between 2.5% and 5.4% [47]). Moreover, it was obtained by using very broad categories as predictors, which suggests that a higher predictive ability could be achieved by more refined analyses that distinguished between subfields and/or specific factors that might influence outcomes. These factors, summarized below, are few and could be tested by future studies.

The probability of a paper to report a positive result depends essentially on three components: 1-whether the tested hypothesis is actually true or false; 2-logical and methodological rigour with which the hypothesis is linked to empirical predictions and tested; 3-statistical power to detect the predicted pattern (because low statistical power decreases the probability to reject the “null” hypothesis of no effect [48]).

Statistical power -which is primarily a function of noise in the data and sample size- is typically low in social, behavioural and biological papers [49], [50], [51], [52], [53], and relatively high in disciplines like physics, chemistry or geology. These latter rarely use inferential statistics at all, either because the outcomes are “yes” or “no” answers (e.g. presence or absence of specific chemical compound in a rock) or because their data have such low levels of noise to make any pattern unmistakable [22], [54]. Based on statistical power alone, therefore, the physical sciences should yield as many or more positive results than the other sciences, which should report more “null” results instead. It follows that the differences observed must be caused by some combination of the other two factors:

### 1-Truth value of the hypotheses tested

Hypotheses tested in biological and social sciences could have a higher probability of being true. This is unlikely to be explained by these sciences having stronger theories than the physical sciences (as discussed in the introduction, these latter have, if anything, a more developed and cumulative “core”), or by these sciences testing more trivial hypotheses (originality and innovativeness are rewarded in all fields of research). More plausibly, the truth value of tested hypotheses could differ (if indeed it does differ) because of two sub-factors:

### 1A-Prior knowledge and beliefs

Scientists in softer sciences might chose their hypotheses based on a greater amount of personal observations, preliminary results, and pure and simple intuition that precede a “formal”, published “test” of the hypothesis. How this might affect the objectivity of research is unclear. On the one hand, accurate prior information increases the likelihood that the tested hypothesis is true and therefore, following Bayesian logic, reinforces the “positive” conclusion of an experiment. On the other hand, scientists' prior beliefs, whether or not they are based on accurate information, introduce an element of arbitrariness and subjectivity in research, and by reinforcing scientists' expectations might also increase their confirmation bias [55].

### 1B-Deepness of hypotheses tested

This has been suggested to reflect the level of “maturation” of a science [56]. Younger, less developed fields of research should tend to produce and test hypotheses about observable relationships between variables (“phenomenological” theories). The more a field develops and “matures”, the more it tends to develop and test hypotheses about non-observable phenomena underlying the observed relationships (“mechanistic” theories). These latter kinds of hypotheses reach deeper levels of reality, are logically stronger, less likely to be true, and are more conclusively testable [56]. This scheme aptly describes the scientific status of ecological studies [40], and might contribute to explain not only the HoS, but also to the differences observed between “pure” and “applied” disciplines, because these latter probably test more phenomenological than mechanistic hypotheses.

### 2-Rigour with which hypotheses are linked to predictions

This can be further subdivided in four sub-factors:

### 2A-Flexibility in definitions, design, analysis and interpretation of a research

In sciences that are younger and/or address phenomena of higher complexity, the connection between theories, hypotheses and empirical findings could be more flexible, negotiable and open to interpretation. This would give scientists more freedom in deciding how to collect, analyze and interpret data, which increases the chances that they will produce a support of the hypotheses they believe to be true [45], [57]. In its earliest stages of development, a discipline or field can be completely fragmented theoretically and methodologically, and have different schools of thought that interpret the same phenomena in radically different ways –a condition that seems to characterize many fields in the social sciences and possibly some of the biological sciences [10], [11].

### 2B-Prevalence and strength of experimenter effects and self-fulfilling prophecies

The biasing effect of researchers' expectations is increasingly recognized in all disciplines including physics [58], [59], but has been most extensively documented in the behavioural sciences [60], [61]. Indeed, behavioural data, which is inherently noisy and open to interpretation, might be particularly at risk from unconscious biases. Behavioural studies on people have an even higher risk of bias because the subjects of study can be subconsciously aware of researchers' expectations, and behave accordingly [25], [26], [62]. Therefore, experimenter effects might explain why behavioural studies yield more positive results on humans than non-humans.

### 2C-Non-publication of negative and/or statistically non-significant results

These can remain unpublished because researchers prefer not to submit them and/or because journal editors and peer reviewers are more likely to reject them [63]. In fields that use statistical inference to test the experimental hypothesis (which, as discussed above, tend to be the “softer” ones), the positive-outcome bias overlaps with a more generic bias against statistically non-significant results (i.e. results that fail to reject the null hypothesis), which is well documented in many disciplines [43]. This latter produces an excess of positive results when the tested effect sizes are medium or large. When effect sizes are very small, however, a pure bias against non-significant results should not affect the direction of the outcome (i.e. both positive and negative supports should be published, as long as they are statistically significant) [48]. In this latter case, therefore, a bias against non-significant results could generate an increase in positive results only if researchers in softer sciences tested more generic hypotheses (for example, “x is *correlated* to y” or “x *influences* y” instead of “x is *positively* correlated to y” or “x *causes* y”), and/or if they adjusted their hypothesis after knowing the results (a questionable practice sometimes defined as HARKing [64]). The publication bias against negative and non-significant results can have several causes. In particular, it is expected to be higher in less developed sciences and in fields where the time-lag between hypothesis formulation and testing is longer, because in such conditions the paucity of conclusive empirical evidence is compensated by a higher confirmation bias and “theory tenacity” of the scientific community [40].

### 2D-Prevalence and strength of manipulation of data and results

Several factors are hypothesised to increase scientists' propensity to falsify research, including: the likelihood of being caught, consequences of being caught, the costs of producing data compared to publishing them, strong belief in one's preferred theories, financial interests, etc…[65], [66], [67], [68]. Each of these factors leads to straightforward predictions on where misconduct is most likely to occur (e.g., in fields where competition is high, replicability is low, conflicts of interest are high, etc…), which very few studies to date have verified empirically. Survey data suggests that outright scientific misconduct is relatively rare compared to more subtle forms of bias, although it is probably higher than commonly assumed, particularly in medical/clinical research [69].

Critics might argue that these results, like previous attempts to measure the hardness of different fields, simply reflect cultural differences between “academic tribes” [30]. However, this study is different from previous ones because it measures a parameter linked to the outcome of research itself. Future studies might show, for example, that a specified discipline has a high frequency of positive results largely because it has a “cultural tradition” of keeping negative results in drawers (or of dropping outliers, or of HARKing, etc…). Such a tradition, however, would have clear and direct consequences for the reliability of the scientific literature in that discipline.

Perhaps the strongest counter-interpretation of these results could be that scientists in different disciplines or fields use the expression “test the hypothesis” in slightly different contexts. For example, sociologists and molecular biologists might use it more when they have positive results, while astronomers and physicists when they have negative results. Although this possibility cannot be ruled out, it seems unlikely to fully explain the patterns observed in this study. Even if it did, then we would have to explain why a certain use of words is correlated so strongly with the hypothesised hardness of different fields and methodologies. In particular, this would suggest that a falsificationist approach to research [70] is applied differently (more rigorously) in the physical sciences than in the biological and social sciences, ultimately supporting the conclusion that the hierarchy of the sciences reflects how research is done.

Papers testing multiple hypotheses were more likely to report a negative support for the first hypothesis they presented. This suggests that the order in which scientists list their hypotheses follows a rhetorical pattern, in which the first hypothesis presented is falsified in favour of a subsequent one. It also suggests that part of the papers that in this study were classified as “negative supports” were in fact reporting a positive result. Since papers reporting multiple hypotheses were more frequent in the social sciences, and particularly in the discipline of Economics and Business, it is possible that these sciences yield more positive results than it appears in this analysis. However, there was no statistically significant difference between disciplines or domains and large differences could be excluded with significant confidence, which suggests that the rhetorical style is similar across disciplines.

A major methodological limitation of this study is the data extraction protocol, because the classification of papers as positive and negative was not blind to the papers' discipline and methodology. Therefore, the confirmation bias of the author himself could not be controlled for. However, parallel analyses on the same sample showed significant correlations between positive results and independent parameters hypothesised to increase scientific bias (Fanelli, submitted). The scoring of papers was completely blind to these latter parameters, which suggests that the proportion of positive results measured in this sample is a genuine proxy of confirmation bias.

Given what sociologists have sometimes written about sociology (e.g. that it is probably the only science where knowledge is truly socially constructed [11]), economists of economics (e.g. that econometrics is like alchemy, with regression analysis being it's philosopher's stone [71]), and psychiatrists of psychology and psychiatry (e.g. that they “pretend to be sciences, offering allegedly empirical observations about the functions and malfunctions of the human mind” [72]), it could be surprising to find any negative results at all in these disciplines. As argued above, this study suggests that such categorical criticisms of the social sciences are excessive. However, at least two limitations need to be considered. First, this analysis is based on the assumption that scientists are generally biased towards positive results, which is well documented [38], [41], but not always true. Scientists will sometimes be biased against the hypothesis they are testing. The frequency with which this occurs might vary by discipline and thus represent a confounding variable. Second, and most importantly, the analysis focussed on papers that explicitly embraced the scientific method and are published in English-speaking scientific journals. However, most of the research published in the social and behavioural sciences is qualitative, descriptive or speculative, and is published in monographs rather than journals, so it eludes the conclusions of this study.

## Materials and Methods

### Data collection

The sentence “test* the hypothes*” was used to search all 10837 journals in the Essential Science Indicators database, which classifies journals univocally in 22 disciplines. When the number of papers retrieved from one discipline exceeded 150, papers were selected using a random number generator. In one discipline, Plant and Animal Sciences, an additional 50 papers were analysed, in order to increase the statistical power of comparisons involving behavioural studies on non-humans (see below for details on methodological categories). By examining the abstract and/or full-text, it was determined whether the authors of each paper had concluded to have found a positive (full or partial) or negative (null or negative) support. If more than one hypothesis was being tested, only the first one to appear in the text was considered. We excluded meeting abstracts and papers that either did not test a hypothesis or for which we lacked sufficient information to determine the outcome.

All data was extracted by the author. An untrained assistant who was given basic written instructions (similar to the paragraph above, plus a few explanatory examples) scored papers the same way as the author in 18 out of 20 cases, and picked up exactly the same sentences for hypothesis and conclusions in all but three cases. The discrepancies were easily explained, showing that the procedure is objective and replicable.

To identify methodological categories, the outcome of each paper was classified according to a set of binary variables: 1-outcome measured on biological material; 2- outcome measured on human material; 3-outcome exclusively behavioural (measures of behaviours and interactions between individuals, which in studies on people included surveys, interviews and social and economic data); 4-outcome exclusively non-behavioural (physical, chemical and other measurable parameters including weight, height, death, presence/absence, number of individuals, etc…). Biological studies in vitro for which the human/non-human classification was uncertain were classified as non-human. Different combinations of these variables identified mutually exclusive methodological categories: Physical/Chemical (1-N, 2-N, 3-N, 4-Y); Biological, Non-Behavioural (1-Y, 2-Y/N, 3-N, 4-Y); Behavioural/Social (1-Y, 2-Y/N, 3-Y, 4-N), Behavioural/Social + Biological, Non-Behavioural (1-Y, 2-Y/N, 3-Y, 4-Y), Other methodology (1-Y/N, 2-Y/N, 3-N, 4-N).

The five-year impact factor of the journal measured by the Journal Citation Reports was recorded for each paper. Impact factors were then normalized by discipline with mean zero and standard deviation one (i.e. z-transformed).

### Statistical analyses

The strength of the association between ranks of hardness and ranks based on the proportion of positive results was tested with Kendall's τ-c, that between ranks of hardness and positive/negative outcome (which is a nominal category) was measured by Cramer's V.

The ability of independent variables to significantly predict the outcome of a paper was tested by standard logistic regression analysis, fitting a model in the form:
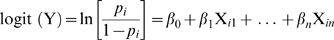
in which *p_i_* is the probability of the *i*th paper of reporting a positive or partial support, and X_1_,… X*_n_*, represent the predictors tested in each regression model, the details of which are specified in the Results section. Statistical significance of the effect of each variable was calculated through Wald's test, and the relative fit of regression models (variance explained) was estimated with Nagelkerke's adjusted R^2^.

Post-hoc statistical power estimations for *X* ^2^ tests assumed Cohen's w = 0.1, 0.3 and 0.5, for small, medium and large effects, respectively. Post-hoc statistical power in logistic regression was calculated for a hypothetical binary variable with bimodal distribution and sample frequency equal to the average sample frequency of all dummy variables in the relevant model (e.g. for a regression with disciplinary domain, the average sample frequency of biological and social sciences). This effect was contrasted to the base-rate probability of the reference category (e.g. for disciplinary domain, the proportion of positive results in physical sciences), assuming no other predictors in the model (i.e. R^2^ = 0). Odds-Ratio = 1.5, 2.5 and 4.5 were assumed to equal a small, medium and large effect, respectively.

All analyses were produced using SPSS statistical package. Power analyses were performed using the software G*Power 3.1 [73].

### Figures

Confidence intervals in the graphs were also obtained by *logit* transformation, using the following equations for the proportion and standard error, respectively:
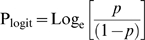


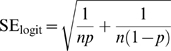



Where *p* is the proportion of negative results, and *n* is the total number of papers. Values for high and low confidence interval were calculated and the final result was back-transformed in percentages using the following equations for proportion and percentages, respectively:
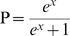


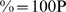



Where *x* is either P_logit_ or each of the corresponding 95%CI values.
